# Idiopathic Purulent Pericarditis Caused by Methicillin-Sensitive Staphylococcus Aureus in an Immunocompetent Adult

**DOI:** 10.7759/cureus.14173

**Published:** 2021-03-29

**Authors:** Deanna L Huffman, Mina Shnoda, Karthik Shankar, Chelsea Peterson, Kushani Gajjar

**Affiliations:** 1 Internal Medicine, Allegheny Health Network, Pittsburgh, USA; 2 Cardiology, Allegheny Health Network, Pittsburgh, USA

**Keywords:** purulent pericarditis, bacterial pericarditis

## Abstract

Introduction: Acute purulent pericarditis is an exceedingly rare entity most often caused by direct intrathoracic contamination or hematogenous spread of a bacterial infection. Mortality nears 100% when left untreated. We present here a rare case of idiopathic bacterial pericarditis caused by methicillin-sensitive *Staphylococcus* *aureus* (MSSA).

Case: A 69-year-old male presented with chest pain and abdominal pain. He was found to have a pericardial effusion and tamponade and underwent emergent pericardiocentesis. Pericardial fluid culture grew methicillin-sensitive *Staphylococcus* *aureus*. The patient required multiple pericardial washouts and was then treated with four weeks of intravenous antibiotics.

Conclusion: While uncommon, clinical suspicion for purulent pericarditis should remain high due to the associated high mortality.

## Introduction

Acute purulent pericarditis is an exceedingly rare entity most often caused by direct intrathoracic contamination or hematogenous spread of a bacterial infection. Idiopathic (spontaneous) cases of bacterial pericarditis are even rarer but few cases have been reported in the literature. Mortality approaches 100% when left untreated. Early detection and aggressive intervention with source control are the keys to improving survival outcomes. As such, the clinician must keep a high index of suspicion for purulent pericarditis when pericardial fluid collection is noted. We present here a rare case of idiopathic bacterial pericarditis caused by methicillin-sensitive *Staphylococcus aureus* (MSSA).

## Case presentation

**Figure 1:**
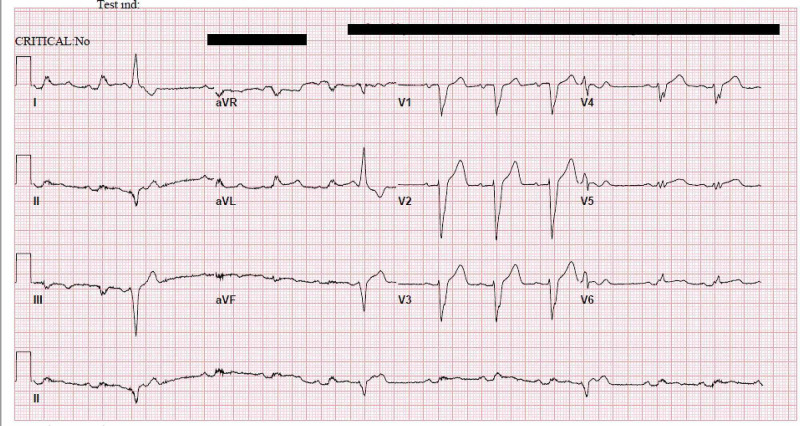
Initial ECG showing sinus rhythm, premature ventricular complexes, and a left bundle branch block. ECG = Electrocardiogram

A 69-year-old male with a past medical history of hypertension and coronary artery disease with remote stenting presented with chest pain and abdominal pain for the past week as well as associated orthopnea. On arrival, the patient had a blood pressure of 86/48 mmHg and a heart rate of 104 bpm; he was afebrile and did not require supplemental oxygen. Electrocardiogram (ECG) showed sinus rhythm with a left bundle branch block and premature ventricular complexes (Figure 1). 

**Figure 2:**
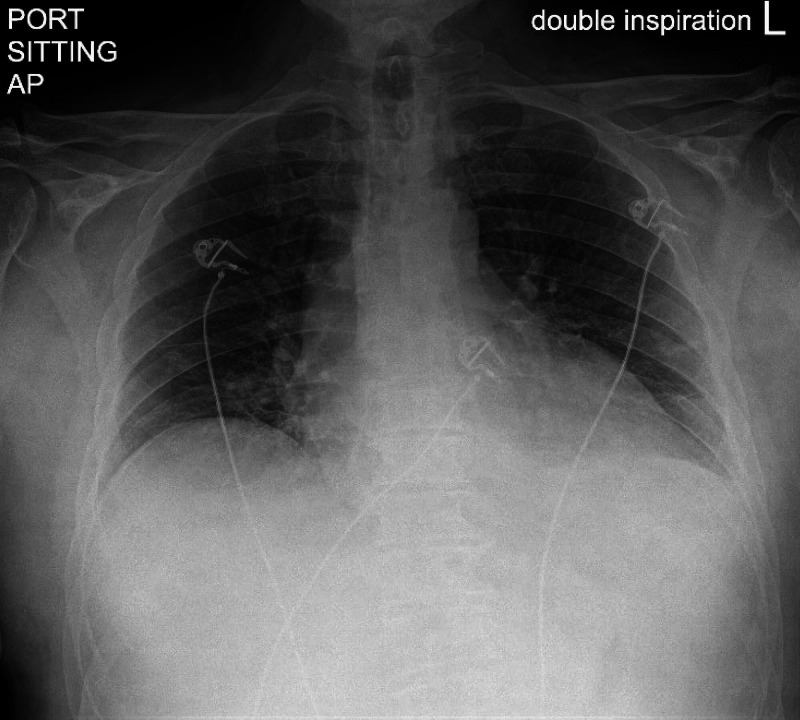
Chest radiograph showing enlarged cardiac silhouette and bibasilar atelectasis.

Physical exam revealed jugular venous distention but no obvious murmur or rubs. Laboratory results were significant for a leukocytosis of 19,000 cells/mm3, elevated C-reactive protein of 25.1 mg/dL, and an elevated erythrocyte sedimentation rate of > 130 mm/hr. Chest X-ray showed an enlarged cardiac silhouette and bibasilar atelectasis but no obvious effusion (Figure 2).

**Figure 3:**
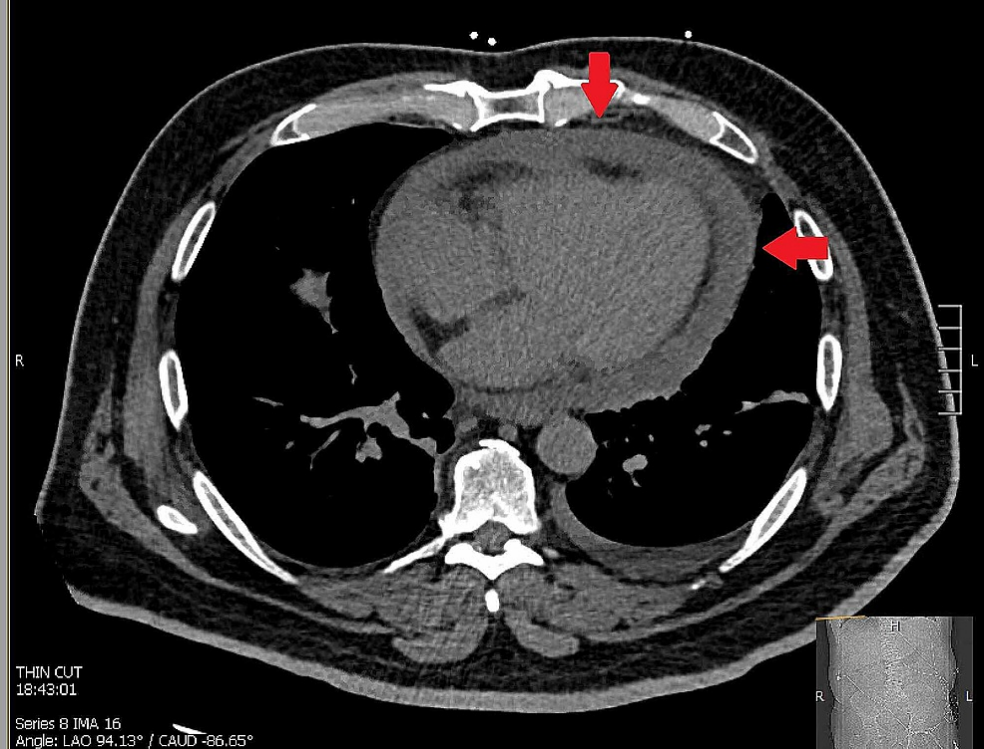
CT scan of the chest with arrows indicating a large, circumferential pericardial effusion. CT = Computed tomography

Computed Tomography showed a large pericardial effusion with moderate pleural effusion but otherwise no intra-abdominal pathology (Figure 3). Bedside transthoracic echocardiography confirmed a large pericardial effusion with tamponade physiology. The patient was emergently taken for pericardiocentesis and 500 cc of straw-colored fluid was aspirated with resulting hemodynamic stability of the patient.

Analysis of the pericardial fluid revealed 2565 nucleated cells (95% neutrophils), glucose of 52 giving a fluid-to-serum glucose ratio of 0.5, and <2000 red blood cells (RBC). Culture of the aspirated fluid grew methicillin-sensitive *Staphylococcus aureus *(MSSA). Interestingly, all blood cultures remained negative and the patient had no history of chest trauma or previous intrathoracic procedures.

**Figure 4:**
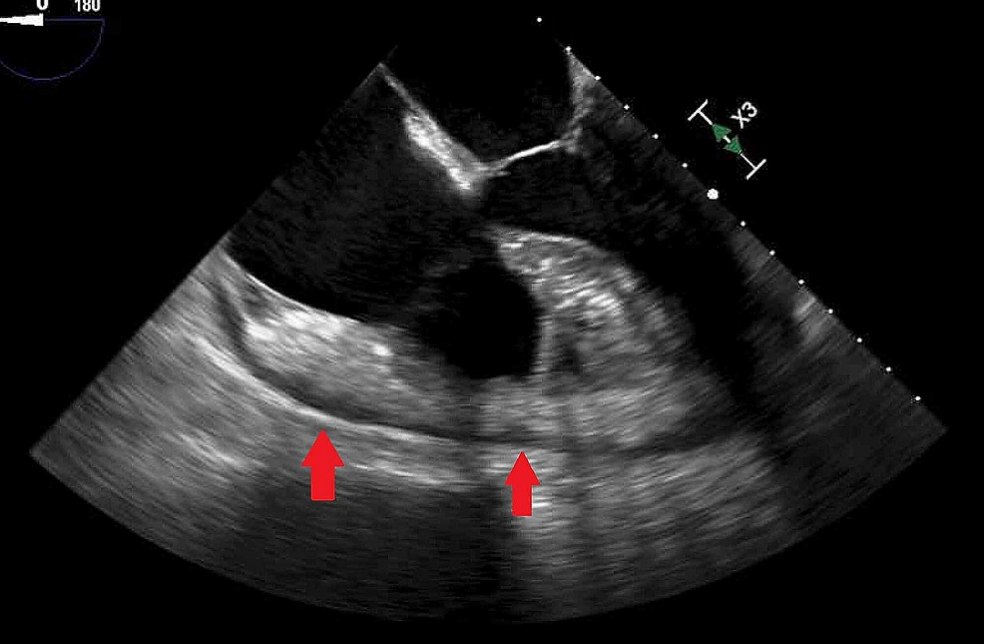
Echocardiogram image performed after pericardial fluid drainage showing small residual effusion (arrows).

The patient had a persistent leukocytosis and again was persistently hypotensive. A transesophageal echocardiogram showed preserved ejection fraction, no valve vegetations concerning for endocarditis, and a reduction in the pericardial effusion with evidence of fibrin products (Figure 4). 

**Figure 5:**
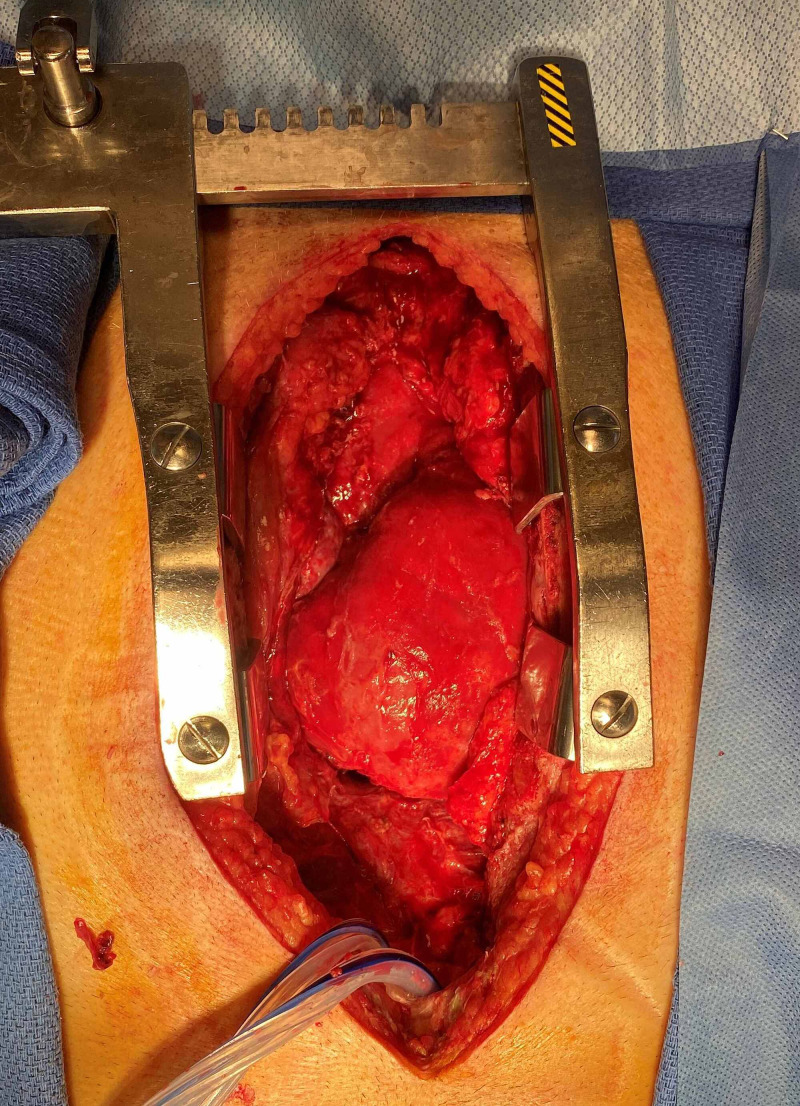
Image of the patient's heart inside the mediastinal cavity, one day after initial pericardial washout was performed. The heart appears edematous but no purulent material has re-accumulated.

It was decided to take the patient's sternotomy and mediastinal washout. Direct examination of the mediastinum revealed extensive inflammation and adhesions of the pericardium and aorta with purulent effusion (Figure 5). Intra-operative cultures also grew MSSA. Biopsy of the pericardium was consistent with fibrosis, chronic inflammation, and fibrinopurulent debris. Cytology was negative for malignancy. The patient was treated with four weeks of intravenous cefazolin from the date of the pericardial washout. The patient required multiple washouts to achieve sterility of the pericardial space.

## Discussion

Bacterial pericarditis is a rare entity that can be rapidly fatal when unrecognized and untreated. The incidence of purulent bacterial pericarditis is reported to be <1%, but rare cases can be found in literature [1]. A 1977 review of 55 patients with purulent pericarditis found on autopsy suggested the disease is most common in the fifth decade and is most commonly caused by gram-negative bacilli, *Staphylococcus aureus* or Streptococcus species [2]. Patients most often present with fever, chest discomfort, shortness of breath, or tamponade. Other etiologies of pericarditis to consider include viral, including HIV-related pericarditis, tuberculosis, malignancy, trauma, and rheumatologic causes.

Pericardial effusion is diagnosed with echocardiography. Bacterial infection is confirmed by culture of the sampled pericardial fluid.

Classically, it has primarily developed in patients with predisposing conditions such as thoracic trauma, intra-thoracic procedures, or the direct spread of a local infection. Immunocompromised individuals are at particular risk. Infections that have been associated with the development of purulent pericarditis include pneumonia, osteomyelitis, meningitis, otitis media, and skin or soft tissue infections [3,4]. Another retrospective review of 33 cases from 1977-1991 revealed pneumonia to be the most common source (18%) [4]. Fungal pericarditis can be seen in immune-compromised patients.

Death from bacterial pericarditis is mostly due to cardiac tamponade or constriction. Systemic antibiotic therapy along with direct rinsing of the pericardial cavity is essential; intrapericardial antibiotics can be helpful but should never be used alone. Open surgical drainage is preferable and pericardiectomy may be required in patients with extensive adhesions, recurrence, or the development of constriction [5]. There are no guidelines on the duration of systemic therapy, but 2-4 weeks is often suggested.

Clinical suspicion should remain high in patients who present with sepsis or tamponade physiology due to the high mortality.

## Conclusions

We present a rare case of idiopathic purulent pericarditis caused by methicillin-sensitive *Staphylococcus aureus*. While uncommon, clinical suspicion for purulent pericarditis should remain high due to the associated high mortality. Early recognition and treatment with appropriate antibiotics and direct source control will hopefully reduce highly fatal late sequelae like restrictive pericardial disease.
